# The role of emotion processing in art therapy (REPAT) intervention protocol

**DOI:** 10.3389/fpsyg.2023.1208901

**Published:** 2023-06-29

**Authors:** Johanna Czamanski-Cohen, Karen L. Weihs

**Affiliations:** ^1^The School of Creative Arts Therapies, Faculty of Social Welfare and Health Sciences, University of Haifa, Haifa, Israel; ^2^Emili Sagol Creative Arts Therapies Research Center, Faculty of Social Welfare and Health Sciences, University of Haifa, Haifa, Israel; ^3^Department of Psychiatry, College of Medicine, University of Arizona, Tucson, AZ, United States; ^4^Cancer Prevention and Control Program, University of Arizona Comprehensive Cancer Center, Tucson, AZ, United States

**Keywords:** art therapy (AT), psycho-oncological care, emotion processing, depression, pain, fatigue

## Abstract

Psychological and physical health are known to improve with emotion processing, which is becoming aware of bodily sensations, accepting them as information that can be translated into emotion concepts and expressing them symbolically and linguistically as emotions. Art therapy utilizes the visual arts for processing emotions to facilitate self-expression and communication with the goal of improving psychological wellbeing. The mental health of individuals coping with and recovering from cancer is known to benefit from art therapy. The purpose of this paper is to describe the development of the role of emotion processing in art therapy (REPAT) intervention, which is an 8 week, one and a half hour art therapy intervention created to target emotion processing as a primary mechanism of change, through which art therapy has the potential to reduce symptoms (i.e., depression, pain and fatigue) of women coping with breast cancer. To obtain this goal we used template for intervention description and replication (TIDieR) and GUIDance for the rEporting of intervention Development (GUIDED) guidelines for intervention development description, with the goal of ensuring successful implementation for clinical and research use.

## Introduction

Globally, there were 2.26 million breast cancer (BC) cases diagnosed in 2020 (Wilkinson and Gathani, 2022) and at the end of 2020, there were 7.8 million women alive who had been diagnosed with BC since 2015, making it the most prevalent cancer (World Health Organization [WHO], 2021). Cancer survivorship is defined as living with the challenges that occur as the result of a cancer diagnosis and treatment (Gage et al., 2011). Many BC patients cope with depression. Stress and negative emotion are normative responses to cancer diagnosis and treatment, but approximately one-third of individuals coping with cancer experience the debilitating consequence of depressive disorders (Mitchell et al., 2011) which have been linked with functional limitation in survivorship (Steiner et al., 2008). Depressive symptoms are clinically elevated in the year following cancer diagnosis in 38% of BC survivors (Stanton et al., 2015; Wu and Harden, 2015). These individuals suffer more physical symptoms, problems with treatment adherence, functional limitation in survivorship (Wu and Harden, 2015) and increased mortality (Cuijpers et al., 2014).

There are several physical symptoms that occur commonly in BC survivors. Pain and fatigue are some of the most prominent symptoms that affect their quality of life (QoL) and wellbeing. A reported 25 to 60% of women develop chronic pain after BC treatment and chronic fatigue is reported in between 30 and 60% of survivors (Gage et al., 2011; Bower, 2014). Physical symptoms co-occurring with emotional distress are some of the most difficult to treat. Cancer survivors with high symptom burden suffer from diminished QoL, which compromises physical, psychological and social functioning (Wu and Harden, 2015).

Emotion processing has been associated with improved physical and psychological health in BC survivors. Emotion processing is comprised of (1) awareness, (2) acceptance, and (3) expression of emotions. Increased emotion awareness occurs when knowledge is transferred from sensorimotor or bodily information to patterns of explicit thought that include conscious processing through language or other symbolic formations, such as visual art (Lane and Schwartz, 1987; Lane et al., 1990). Low levels of emotion awareness are associated with somatoform disorders (Subic-Wrana et al., 2010). Acceptance of emotion is an emotion regulation strategy in which individuals embrace an attitude of being accepting, friendly, and nurturing toward their feelings (Politi et al., 2007; Weihs et al., 2008). Acceptance of emotion has been associated with experiencing less fear, catastrophic thoughts, avoidance behavior and better recovery from negative affect as compared to suppression of emotion. Women coping with BC who were less accepting of their emotions also report greater distress (Politi et al., 2007) and sickness symptoms (Reed et al., 2016). Emotion expression refers to the extent to which feelings are intentionally (Kring et al., 1994) (mainly verbally) and non-intentionally (Collier, 2014) (body language, facial expressions) conveyed to others. Increased emotion expression and reduced avoidance have long been associated with improved wellbeing (Stanton et al., 2002; Weihs et al., 2008; Giorgio et al., 2010; Bardeen et al., 2013). Increased emotion expression is also associated with improved psychological and physical adjustment to BC (Rost et al., 2012; Hoyt et al., 2014). These components of emotion processing are promising as potential mechanisms through which art therapy may improve physical and psychological health in BC survivors.

There are ethno-cultural differences in response to cancer diagnosis. Women from traditional backgrounds, in which there is an emphasis on collectivism as opposed to individualism and a reliance on religion as a major coping strategy, may respond differently to cancer diagnosis and treatment than do more modern/secular women. Women from traditional backgrounds may see cancer diagnosis as fate and fear stigma related to exposing their diagnosis. Furthermore, out of fear of their loss of role in the traditional family, women may not express their distress openly, which leaves them at risk for loneliness and not receiving help for their symptoms (Stanton et al., 2000; Low et al., 2006; Rost et al., 2012). BC survivors from ethnic minorities report poorer social, emotional, spiritual and physical quality of life (Azaiza and Cohen, 2006, 2008). Since expression of emotion and venting is distressing for some ethnic minorities (Goldblatt et al., 2016), art making and the use of metaphors for emotion processing may be less distressing and more helpful in reducing symptoms and increasing quality of life (Goldblatt et al., 2013; Miller et al., 2015).

Art therapy interventions encourage emotion processing. Art therapy is a form of psychotherapy that involves the use of visual artmaking (drawing, painting, sculpting, collage, etc.) for expression and communication within a safe and supportive relationship, in a therapeutic setting. Art therapy has been well documented in cancer settings to alleviate psychological symptoms and reduce physical complaints (Monti et al., 2006; Nainis et al., 2006; Öster et al., 2006; Svensk et al., 2009; Thyme et al., 2009; Slayton et al., 2010; Archer et al., 2015). In a qualitative study, women with BC reported that art making was helpful through increased access to emotional content and its expression (Collie et al., 2006). The above-described literature demonstrates the extensive clinical and research documenting the benefits of art therapy with cancer survivors. However, the literature does not describe in detail the interventions conducted. Thus, one of the goals of this paper is to provide both clinicians and researchers with a detailed account of the intervention development and implementation for the purposes of replication and further research, and more importantly, the accurate implementation of the REPAT intervention with BC survivors.

Intervention development is a complex process through which clinicians attempt to understand their client’s needs and develop ways to intervene so they can experience meaningful behavioral, cognitive, and emotional changes to improve their psychological and physical health, and their general wellbeing. This paper describes the rationale, model, and content of the intervention that we designed, which we call the role of emotion processing in art therapy (REPAT) intervention. We chose to use the template for intervention description and replication (TIDieR) checklist for this purpose, as it is helpful in assuring that interventions are described in ways that other clinicians and researchers can implement and study successfully (Hoffmann et al., 2014). We are also following the GUIDED intervention development reporting items, as it builds on the TIDieR template and was developed through a more recent census-based approach (Duncan et al., 2020).

### The “REPAT” intervention (TIDieR item two)

#### Rationale

Role of emotion processing in art therapy (REPAT) (TIDieR item one) intervention was created by the authors of this paper to target emotion processing as a primary mechanism of change through which art therapy has the potential to reduce symptoms of women following the experience of coping with BC. The purpose of this paper is to describe the development of the intervention, which is based on the clinical experience of both authors, who are an art therapist and psychiatrist, respectively, with extensive clinical and research experience in psycho oncology. In addition, the intervention development is based on the results of previous qualitative and quantitative research on the effect of art therapy with cancer patients in general and BC survivors, specifically (GUIDED item one).

#### Theoretical framework

The bodymind model is a developmental theoretical model of the mechanisms through which art therapy potentiates salutary gains (Czamanski-Cohen and Weihs, 2016). Furthermore, the bodymind model offers validated tools through which the proposed mechanistic changes can be measured in empirical studies. This theoretical standpoint assumes there are specific mechanisms activated through art therapy, and through these mechanisms art therapy has the potential to improve psychological and physical health (GUIDED item six). At the time of this writing, to the best of our knowledge, the bodymind model is the only model of art therapy offering potential mechanisms and ways to measure them. Thus it was chosen as a framework for the design of the intervention, which was designed to activate the mechanism of emotional processing, deemed potentially important for the reduction of symptoms in BC survivors. The treatment protocol also derives elements from the application of focusing to art therapy (Rappaport, 2008) for the purpose of body awareness and focusing (“being friendly, accepting, non-judgmental and welcoming to one’s inner felt sense”) as well as some interventions from the Cognitive Behavioral Art Therapy (CBART), which is a six session cognitive behavioral intervention that utilizes art making for stress reduction and self-expression and was developed for work with individuals coping with chronic illness (pain and cancer), and has been adapted for working with women coping with post-partum depression and infertility (Czamanski-Cohen et al., 2014) (GUIDED item seven).

#### Aims

The target population of the originally designed REPAT study are BC survivors, however, we believe the intervention in its current form can be easily utilized with other cancer survivors (GUIDED item three). In addition, our team is currently adapting the intervention for work with individuals coping with inflammatory bowel disease as well as individuals coping with social anxiety. The outcome targets of the intervention are reductions in symptoms of depression, pain and fatigue as well as increases in emotion processing. The art therapy session includes (i) an introductory period in which the therapist engages with participants to establish rapport and begins to understand participants’ current state of mind, (ii) an art making period which entails much of the time, and (iii) a processing period in which the art made is reflected upon and discussed. We hypothesize that increased Emotion Processing is a primary mechanism through which art therapy effects psychological and physical symptom reduction in BC survivors and thus we designed the REPAT intervention to test this hypothesis.

## Materials and equipment

### Materials used in the intervention delivery or in the training of intervention providers (TIDieR item three)

The art therapist provides a variety of art materials, that are either presented in the middle of the table, if the intervention is being delivered face to face, or mailed to each participant, if the intervention is being delivered virtually through an internet-based program, such as Zoom. We specifically kept the list of materials simple to be able to conduct the intervention in hospital conference rooms, which at the time of the design of the study, were the rooms we thought we would be using for the intervention groups. The following is a list of materials that we used in the intervention, and recommend to others. However, we also believe that flexibility may be required when adapting the intervention for use with other populations and settings. #1: a 24 color package of Panda oil pastels (Royal Talens, Apeldoorn, Netherlands). We chose these pastels as they are made with pure pigments, mineral oils, and wax binders for a soft and smooth laydown with no dust, and they work on various platforms. The color selection in the 24-color set includes: white (100.5), light yellow (201.5), deep yellow (202.5), lemon yellow (205.5), yellow ochre (227.5), orange (235.5), vermillion (311.5), scarlet (334.5), deep rose (362.5), burnt umber (409.5), burnt sienna (411.5), sepia (416.5), ultramarine (504.5), Prussian blue (508.5), turquoise blue (522.5), violet (536.5), red violet (545.7), blue violet (548.5), Phthalo blue (570.5), permanent green medium (614.5), sap green (623.5), fir green (654.5), phthalo green (675.5), and black (700.5), #2: sets of 24 color Birello double tip felt pens (Carioca, Italy), #3: sets of 24 colorpeps colored pencils [Manufacture d’Articles de Précision Et de Dessin (Maped), France], #4: HB pencils, #5: pencil sharpeners, #6: erasers (Milan, Spain) and #7: A4 printer paper (Kravitz, Israel).

At the end of the intervention, participants received a package of 12 color Panda oil pastels (Royal Talens Apeldoorn, Netherlands) which includes: white (100.5), lemon yellow (205.5), orange (235.5), vermillion (311.5), carmine (318.5), light blue (501), ultra marine (504.5), light green (601), green, yellow ochre (227.5), light brown (401), and Black (700.5) to take home along with a drawing block with 35 cm × 50 cm, 240 gram weight paper (Metro, Israel).

## Methods

### Procedures, activities, and processes (TIDieR item four)

The REPAT intervention is an 8 week group intervention comprised of eight one and a half-hour weekly sessions. The art therapist initiates metaphorical or concrete discussions with clients in order to facilitate their transition from one core therapeutic process to the next. For example, after creating a safe environment through the triangular relationship (client, therapist and art) the client is encouraged to engage their self through art making. In this process the therapist is supportively present. The client may make new discoveries about their self through the movement of emotional material from the implicit to explicit arena.

The therapist (based on their theoretical approach and the needs of the client) may remain in the metaphor while discussing the content of the art with the client (art as therapy, phenomenological approach) or they may use a more explicit conversation about the content and its meaning (art psychotherapy). If these discoveries are distressing, the client may need to receive additional support by returning the focus to strengthen the attachment relationship with the art therapist. The client, with guidance of the art therapist can use the content of the art expression to take a reflective stance and observe multiple perspectives thus enhancing meta-cognitive processes related to increased physical and emotional health (Fledderus et al., 2010, 2012; Fonagy et al., 2011; Lengacher et al., 2015). After the expression of somato-emotional and cognitive knowledge, the art therapist can facilitate engagement in reflective or perspective taking processes, or in broader metacognition (thinking about thinking).

### The development of the reflective self: social understanding

Through the appreciation of the reasons behind the actions of caretakers and siblings the child learns to acquire a representation of her own desires, wishes and mental states, coined intentionality. Playful interpersonal interactions through art making provide the basis for additional growth in intentionality for adults. They will develop self-agency if they permit: (a) the registration of perceptions, thoughts, and emotions as causes and consequences of action and (b) the contemplation of these mental states without fear.

Art therapy sessions start with a 10-min rapport building discussion and continue with a brief relaxation exercise followed by 40 min of art making in a calm and supportive environment. Art materials are on the table and after the art therapists provides a brief explanation of the use of the materials, participants were encouraged to explore and experience as they wish. The art therapist encourages participants to refrain from conversation and instrumental music is played to encourage introspective experiences. The role of the art therapist is to encourage a non-judgmental and exploratory approach to artmaking in which the process is emphasized over product. The art therapist fosters this approach by creating an atmosphere that is calm and by remaining tuned-in to the verbalizations and body language of participants. If needed she can provide individual attention that is geared toward neutralizing concerns regarding performance during the art making. This approach is defined as providing a “Third-hand” (Kramer, 2001): assisting in problem solving and dilemmas related to the art making process. The sessions end with 30 min of processing and discussion in which the art therapists request each participant to share and briefly present their work, to which group participants respond and/or provide support. The art therapist reminds group members to be respectful and non-judgmental toward other participants and themselves when they share their artwork.

The following is a detailed description of each session, each of which has its own objective (delineated in Table 1):

**TABLE 1:** The REPAT intervention.

	Treatment goals	Intervention
Session 1: getting familiar and feeling safe	Rapport building Contract building Getting familiar and comfortable with the art materials	The concept of safe place will be introduced to the group. Motivation for exploration via art and remaining open to new experiences. The concept of the group will be introduced, expectations, group rules, homework introduce the art materials and art making from a non-judgmental stance and the importance of process over product. Participants are instructed to create a safe place via art.
Session 2: exploratory art making	Provide an experiential encounter with the art making process	A 10-minute psychoeducation presentation on the nature of emotions at the beginning of the session. The framework of open studio in which a specific topic is not provided, and participants are encouraged to explore the art materials
Session 3: engaging the emotional self	Participants are asked to introduce themselves to the group. Participants are encouraged to continue learning on the nature of emotions and increase awareness of emotion responding patterns.	Participants are asked to create a drawing about an emotion.
Session 4: image transformation	Participants will learn how to identify the location of distress in the body, increase distress tolerance and increase cognitive flexibility and reframing.	Participants are asked to create an image of somethings that is distressful to them and sit with it for a while. Afterwards they are asked to create an additional drawing that changes one element of the distress drawing, a feature, a color, a shape, or just a change in composition. The images are discussed among group members and implications for real life situations are discussed.
Session 5: open studio	Help participants identify how they react and respond to their emotions and help them increase their awareness of emotional experiences.	Participants are encouraged to engage freely with the art materials using artmaking to identify feelings and experiences with awareness that feeling are not reality.
Session 6: reframe	Increase cognitive flexibility and reframe.	Clients are requested to draw two sides of a current conflict in their life. It can be something small, like deciding where to go for lunch, or something large, like which treatment to engage in. All conflicts are welcome, but it should have significance to its creator and be something that they are struggling with and would like to learn more about. After the drawing period, clients are requested to look at both options and examine their sensations, feelings, and thoughts about each. Through creating 2 pictures participants will be able to view a situation from 2 points of view and identify more than one option for coping with the conflict they presented.
Session 7: body image	Increase interoceptive awareness and assist in processing emotional content from implicit experience to explicit expression. Increase distress tolerance.	Participants are requested to create art using body outline templates as a framework for art making.
Session 8: summary	Create art that is a summary of their experience and then engage in and review their achievements and encourage incorporating what was learned in day-to-day life after the intervention is over.	A summary of all that has been experienced and clients is provided with a letter that summarizes their progress. Each client receives a package of oil pastels and blank journal for the encouragement of their continued process at home.

#### Session one

##### Getting familiar and safe

The group leader will speak about motivation for exploration via art and remaining open to new experiences. This includes the awareness that our emotions are information that is provided to us about how our needs and wants are being fulfilled. At this point the discussion is more general and is focused on reducing judgmental self-talk and paying attention to somatic experiences and using art materials to explore with the purpose of becoming more self-aware. The art therapist introduces the art materials and art making from a non-judgmental stance and will emphasize the importance of process over product. The concept of safe place will be introduced to the group and members will be asked to create a safe place via art (Czamanski-Cohen et al., 2014). The goals of this session: rapport building, contract building, and getting familiar and comfortable with the art materials.

The concept of the group is introduced by the art therapist, to include expectations for what will be gained from participation in the 8-week protocol, along with the group rules (arriving on time, respecting all members, keeping information about other group members private, etc.). The art therapist introduces the art materials and art making from a non-judgmental stance and the importance of process over product. The concept of safe place is then introduced to the group through a guided imagery exercise through which the art therapist invites the participants to imagine a place (real or imaginary) that they find beautiful and that encourages a sense of safety. The participants are guided by the art therapist to activate their five senses and imagine the sights, sounds, smell, taste of the air, and sensation on their skin of their chosen safe place. Participants are then instructed to create a safe place either concrete or abstract using the art materials. They are asked to remain open to exploration via art while also remaining open to new experiences in general.

#### Session two

Art making will be exploratory in the framework of open studio in which a specific topic is not provided, and participants are encouraged to explore the art materials. The goal of this session is to provide an experiential encounter with the art making process. There will be a 10-min psychoeducation presentation on the nature of emotions at the beginning of the session (see Appendix).

After a 10-min rapport building exercise that invites checking in by each group member, the art therapist conducts a brief 10-min psychoeducation presentation on the nature of emotions. We utilize the constructivist theory of emotions through which emotions begin as a somatic experience that is translated and defined as a specific emotion based on our past experiences (for additional reading see Barrett, 2017). The participants are requested to “play” with the materials and create whatever they would like, in the framework of an open studio in which a specific topic is not provided. Participants are encouraged to explore the art materials and are encouraged to focus on their process.

#### Session three

Group members will introduce themselves to the group by making a drawing about an emotion. The goal of this session is for participants to continue learning about the nature of their emotions and to increase awareness of their emotion responding patterns. Participants are asked to choose an emotion to draw as an introduction of themselves to the group. The art therapist encourages the participants to continue learning about the nature of emotions and to increase awareness of their emotion responding patterns. Participants are asked to create a drawing about an emotion.

#### Session four

The art therapist requests the participants to create an image of something that is distressful to them and sit with it for a while. Then, they are requested to create an additional drawing that changes one element of the distress drawing, a feature, a color, a shape, or just a change in composition. The images are discussed among group members and implications for real life situations are discussed (Czamanski-Cohen et al., 2014). The goal of this session is twofold- to identify the location of distress in the body, increase distress tolerance, cognitive flexibility, and reframing. Participants are asked to create an image of their distress and sit with it for a while. Afterward they are asked to create an additional drawing that changes one element of the distress drawing, a feature, a color, a shape, or just a change in composition. The images are discussed among group members and implications for real life situations are discussed.

#### Session five

Due to the intensity of session four, which can be seen as a pinnacle of the REPAT intervention, session five is an open studio, in which participants can use art, however, they choose. This session is open studio in which participants are encouraged to engage freely with the art materials. The goal of this session is to help participants identify how they react and respond to their emotions and bring an awareness of emotional experiences and learn the skill of “Clearing the Space”-using artmaking to identify feelings and experiences without the need to identify with them. This means that participants are asked to become in tune with somatic experiences and express them on paper with art material in an exploratory manner. This externalization of emotional material enables both a distancing and reflection upon this material, and an awareness of these emotions as informative and transitory (Rappaport, 2008).

#### Session six

Participants are requested to draw two sides of a current conflict in their life. It can be something small, like deciding where to go for lunch, or something large, like which treatment they want to receive. All conflicts are welcome, but the one chosen as a focus for this activity should have significance to its creator and be something with which she is struggling and about which she would like to learn more. After the drawing period, clients are requested to look at both options and examine their sensations, feelings and thoughts about each. Their art, thoughts and feelings are shared with the group. The goal of this session is to increase cognitive flexibility and to reframe ones perspective on the conflict. This means that through creating two pictures participants will be able to view a situation from two points of view and identify more than one option for coping with the conflict they presented. Clients are requested to draw two sides of a current conflict in their life. It can be something small, like deciding where to go for lunch, or something large, like which treatment to receive. All conflicts are welcome, but it should have significance to its creator and be something with which they are struggling and about which they would like to learn more. After the drawing period, clients are requested to look at both options and examine their sensations, feelings, and thoughts about each. Through creating two pictures, participants will be able to view a situation from different points of view and identify more than one option for coping with the conflict they presented. The goal of session six is to increase cognitive flexibility and the ability to reflect upon and to reframe a difficult situation.

#### Session seven

##### Body image

In this module participants create art using body outline templates as a framework for art making. The goal of this session is to increase introceptive awareness and assist in processing emotional content from implicit experience to explicit expression. It also aims to increase distress tolerance.

#### Session eight

##### Summary

This session will be a summary of all that has been experienced and clients will be provided with a letter that summarizes their progress from the perspective of the art therapist. Each client will receive a package of oil pastels and blank journal for the encouragement of their continued process at home. The participants are asked to draw an image that reflects what they have obtained during the part 8 weeks, and what they wish for themselves for the future. The group then conducts a portfolio review where they look at all the art created in the past 8 weeks. The goal of this session is to review achievements and encourage incorporating what was learned in day-to-day life after the intervention is over.

### Expertise and background of program facilitators (TIDieR item five)

Program facilitators are experienced Master of Arts level art therapists who participate in a 4–6 h training program. Details of the training module can be obtained by contacting the corresponding author of this paper. It is recommended that the art therapists have previous experience working with adult cancer patients and survivors and are aware of the common issues with which these individuals are coping. During the implementation of the intervention, it is recommended that the art therapists receive supervision from a senior clinician who is well versed in the REPAT intervention.

### How, when and how much (TIDieR items six and eight)

The intervention was designed to be implemented face to face in a group setting for 1 h and a half, each week for 8 weeks. See previous section for details of how, when and how much.

### Location(s) where the intervention occurred, including necessary infrastructure or relevant features (TIDieR item seven)

**FIGURE 1:**
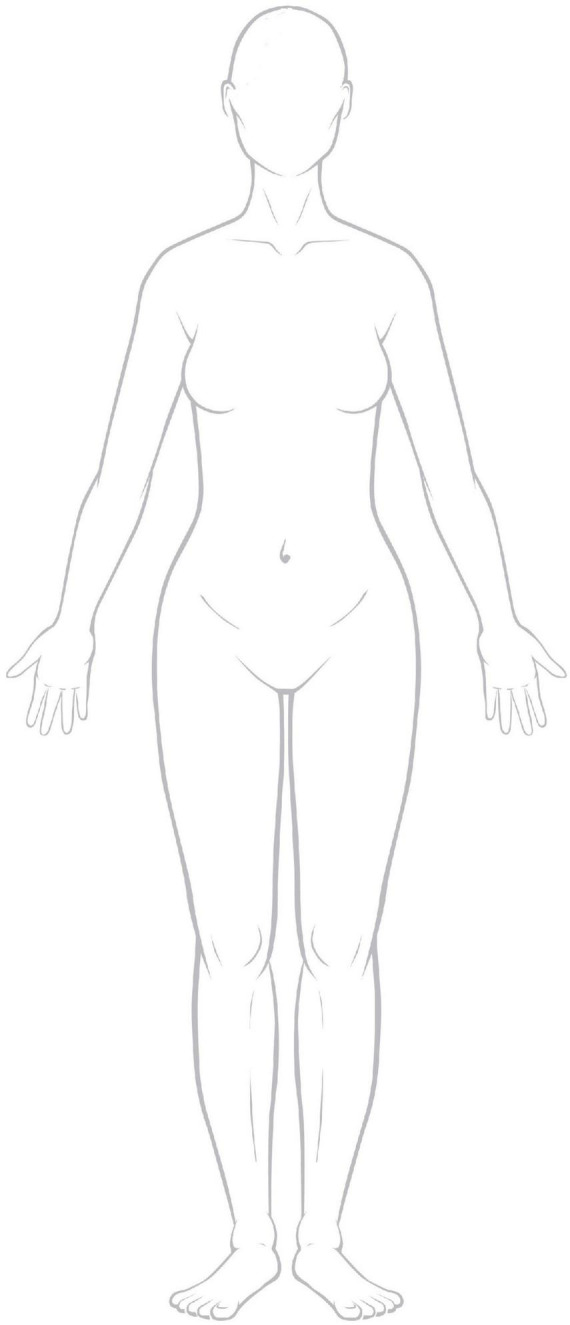
Template of body outline used in session seven.

The intervention was designed to be implemented in hospital meeting rooms, in lieu of the availability of a preferred art therapy studio. The minimal necessities to conduct the intervention are a room with a table in the middle and chairs, with enough space for 10 participants and the art therapist. Lighting needs to be bright enough for participants to clearly see what they are creating. The art supplies mentioned above should be placed in the center of the table so that each type of material is easily accessible to all participants. A photocopy of a human figure is needed for session seven. Figure 1 shows the human shape used in our intervention.

### Tailoring (what, why, when, and how) (TIDieR item nine)

During the COVID-19 pandemic, we had the opportunity to pilot and implement the study online, in a virtual setting through which each participant logged on to an online platform, such as Zoom (Zoom Video Communications, Inc, 2020). Due to the technical needs of conducting the intervention online, we had an assistant present to assist participants with technical difficulties, or to support the group if the art therapist needs to provide additional support to a group member. Furthermore, we extended the time for the online groups to 2 h instead of one and a half hours to accommodate adjustments to technical and logistic issues that were inherent in the use of the online platform. We found this was an important adaptation as time seemed to flow differently online, and more time was needed for the checking in as well as the summation portions of each session.

To adapt to an online setting, we mailed art supplies, paper and the photocopy of the body outline to the participants several weeks before the intervention began. We were also able to provide tablet computers with data service and a headset for participants who did not have access to the internet (GUIDED item ten).

### Modifications, adherence, and fidelity (TIDieR items ten and eleven)

We utilized a fidelity chart (Table 2) completed by the art therapist after each session. If fidelity in any area was below 80% the first author conducted an additional supervision session with the art therapist who conducted the session to determine how fidelity could be improved for increased adherence to the protocol.

**TABLE 2:** Fidelity assessment.

	1 not at all	2 a little bit	3 neither yes or no	4 quite a bit	5 very much so	Not applicable
(1) Was there a sense of calm in the room?	1	2	3	4	5	N/A
(2) Did you feel like you were able to support the participants?	1	2	3	4	5	N/A
(3) Were the participants deeply engaged in art making?	1	2	3	4	5	N/A
(4) Was the session divided in to a 10-min intro, 60 min art making and 20 min discussion?	1	2	3	4	5	N/A
(5) Was the art making done with minimal conversations?	1	2	3	4	5	N/A
(6) Was the group discussion respectful and safe?	1	2	3	4	5	N/A

An additional strategy that is preferred, however, not always feasible, is to have an experienced trained art therapist observe the newly trained art therapist while they implement the intervention for the first time. Following being observed for the first time implementing the intervention, and receiving supervision and direct feedback, a newly trained art therapist is observed for a second time and, if fidelity to the protocol is achieved, they can be considered “certified” in the REPAT intervention and continue to provide fidelity reports for her own interventions.

### Strategies to improve and maintain fidelity (TIDieR item eleven)

To improve and maintain the fidelity of the intervention, it is recommended to conduct fidelity checks, such as the one provided in Table 2. The fidelity checks ensure the main elements of the protocol regarding the division of time, art making and processing discussion are occurring in the intervention provided.

## Results

In the following section we describe and illustrate the results of the REPAT intervention, using examples from participants’ artwork and quotes of participants regarding their experience that we collected following the pilot study of the online intervention. Furthermore, we describe where the intervention has been implemented and how it was received.

**FIGURE 2:**
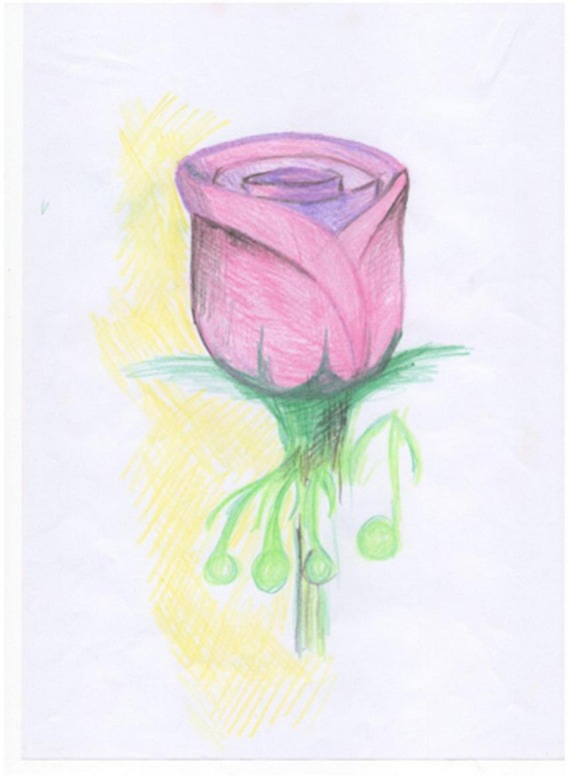
Drawing of participant from session one.

**FIGURE 3:**
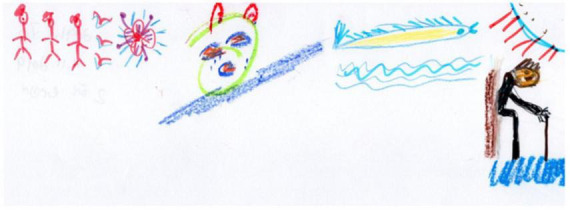
Drawing of participant from session two.

**FIGURE 4:**
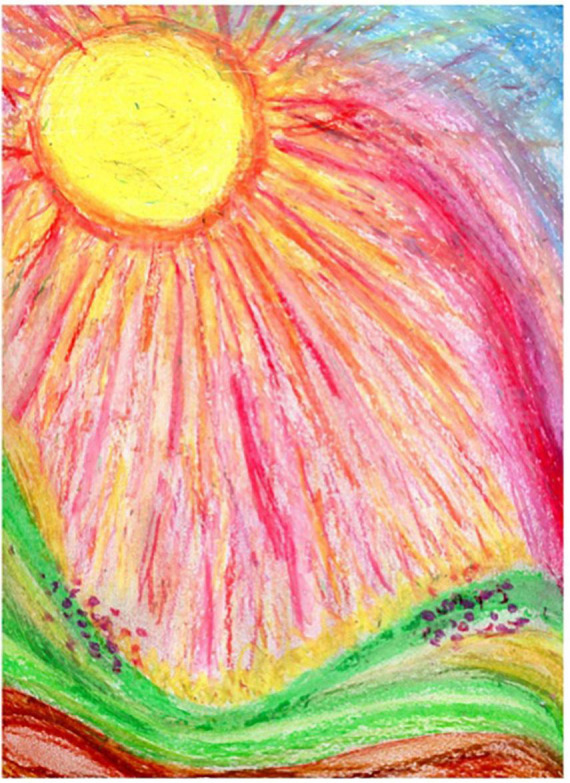
Drawing of participant from session three.

Session one of the REPAT intervention is a “safe place” intervention. Figure 2 is an example of a safe place created by a group member who stated that “growing flowers” is her safe place, and this activity makes her feel connected. Session two focuses on exploratory art making which provides an experiential encounter with the art making process. Figure 3 is an example of art made by a participant in session two in the REPAT study. Session three deals with engaging the emotional self. Figure 4 is a drawing of a participant in the REPAT study who stated that she was drawing about happiness, and that she rarely gave herself enough time to have in depth experiences with herself. She stated that she identified with emotions created by other group participants such as anger and feeling shaken by the experience of cancer.

**FIGURE 5:**
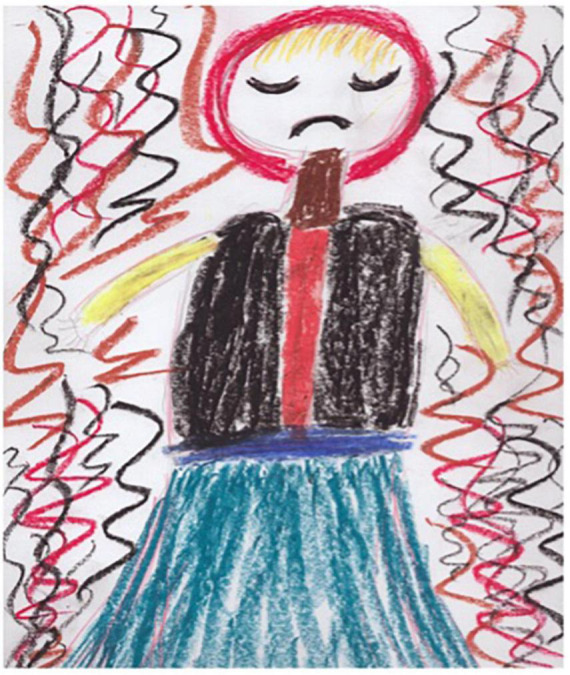
First drawing of participant from session four.

**FIGURE 6:**
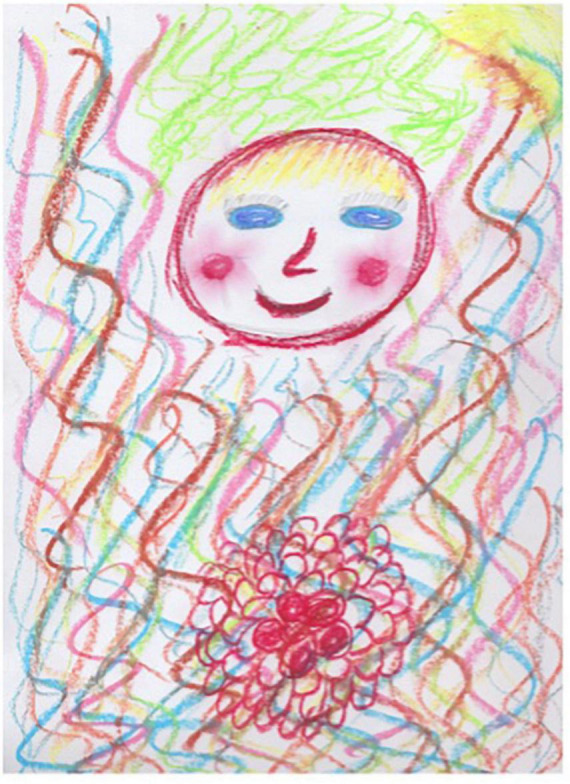
Second drawing of participant from session four.

**FIGURE 7:**
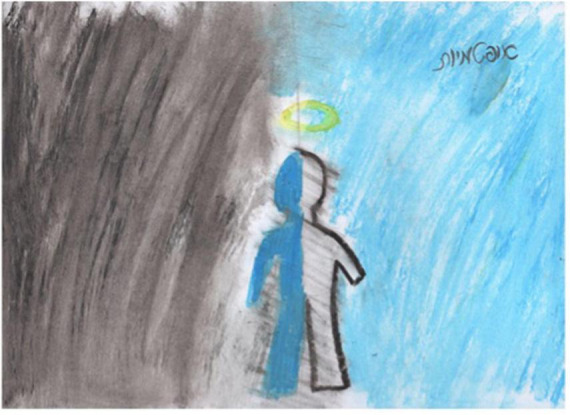
Drawing of participant from session five.

Session four is an image transformation exercise. Participants are guided by the art therapist to identify the location of distress in the body, and get in touch with this sensation for several moments with the goal of increasing distress tolerance. The goal of the session, other than distress tolerance is increasing cognitive flexibility and reframing., that occurs by creating the second drawing and creating a shift in the experience of distress, initially on paper, and eventually as a whole-body experience. This participant drew her physical pain in Figure 5. The drawing depicts the pain surrounding her and splitting her upper body in to two parts. In Figure 6, we see that the participant removed the body from the drawing and these are replaced by colorful squiggles and a flowerlike shape. She stated “When I am in pain, I can’t talk to anybody, or think of anything. I don’t even have words to describe it. When the pain goes away, I can communicate, I can think.” She later responded to another participant, saying “It feels really good to get all of our feelings out on the paper, right?” The goal of this session is to continue to help participants identify how they react and respond to their emotions and bring an awareness of emotional experiences. Participants are encouraged to engage freely with the art materials using artmaking to identify feelings and experiences with awareness that feeling are not reality. Figure 7, is a drawing in which a participant is split in half so that that the left side represents all the “filth” in her life. She shared with the group, that even before her cancer diagnosis, she had to deal with her husband being in jail for 12 years. She stated that despite all her difficulties, she was able to maintain optimism, that is depicted on the right side of the drawing, and the word “optimism” is written in Hebrew in the right upper corner of the drawing. She received feedback from the group that it was her optimism that enables the figure to stand stable and strong amid these difficulties, and she thanked the group for the feedback, stating that she was not previously aware of the fact that being optimistic had such a positive effect on her.

**FIGURE 8:**
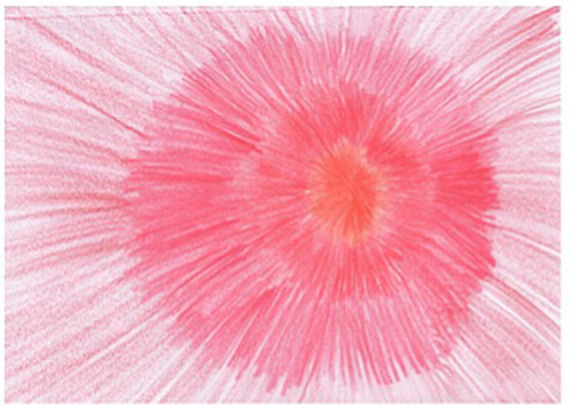
First drawing of participant from session six.

**FIGURE 9:**
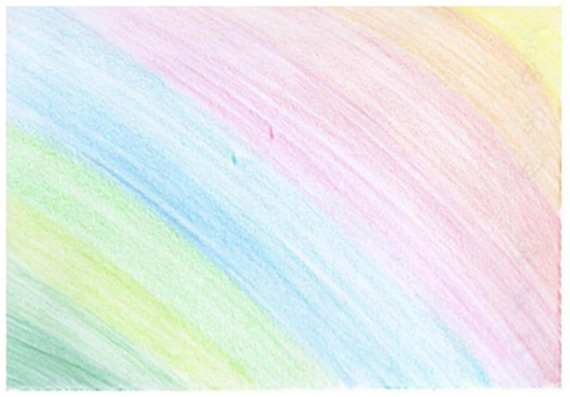
Second drawing of participant from session six.

Session six is designed to deal with the concept of reframe. In Figure 8 a participant depicted how she feels stressed at her current job, and in Figure 9, how relaxed she felt while she was going through cancer treatment, and she took time off, and was able to spend time with her family. During that time, she promised herself not to return to her stressful position, however, now she understands that it is more complicated than she previously perceived.

**FIGURE 10:**
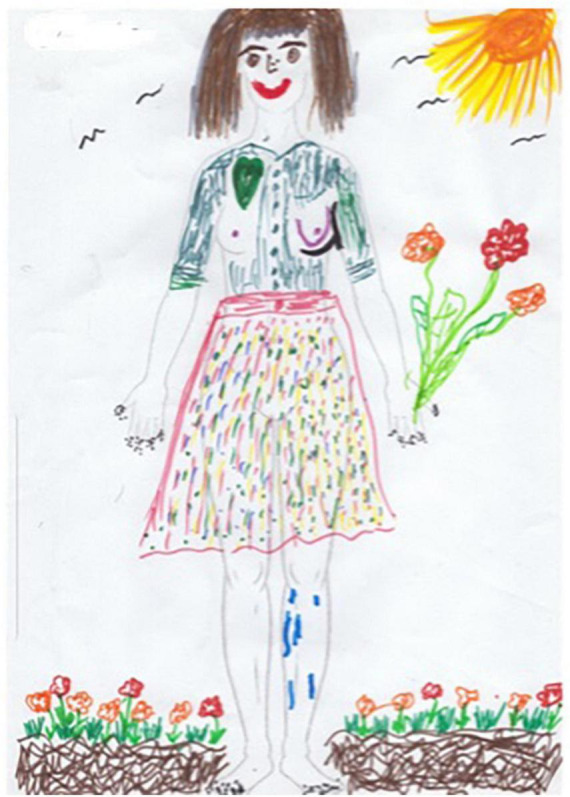
Drawing of participant from session seven.

Session seven is focused on body image using body outline templates as a framework for art making, however, they are instructed to use the template, or more than one template in any way they desire (Figure 10). A participant in the REPAT study shared that she drew herself as she envisioned in her mind’s eye, as healthy. She drew herself wearing a skirt which she found strange because this is not what she usually wears when she feels well and wore skirts for chemotherapy because it was more convenient to place the IV when she was in a skirt. She drew her surgery on the right breast. She stated that she drew her hair, which was very long before cancer treatment and she is amazed at how fast it is growing back. She remembered that she cut off two braids that she intended on donating but ended up making a wig for herself out of them. She added the sun, stating that the sun is very important to her, she wants to return to her life.

**FIGURE 11:**
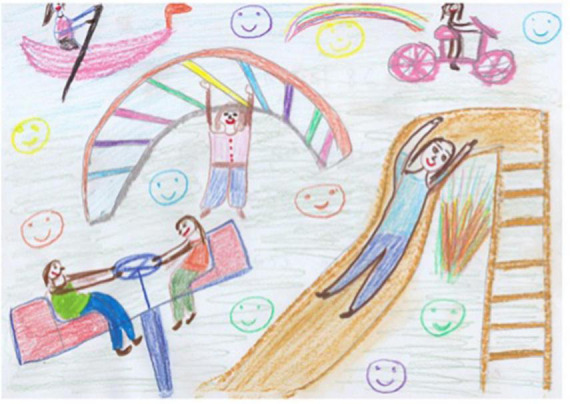
Drawing of participant from session eight.

Session eight is the last session of the REPAT intervention and is intended to be a summary of the participant’s experiences and is designed to engage in and review their achievements and encourage incorporating what was learned during the sessions in their day-to-day life after the intervention is over. The art therapist writes a letter to each participant that summarizes all that has been experienced and their progress. Participants are asked to create a drawing that creates a summary for themselves and that includes what they wish for themselves going forward. A participant in the REPAT study stated: “I can’t believe its already over. A lot of interesting things have been happening to me in the past few weeks, I can feel newfound strength. Last week’s drawing released something in me, like the bottle I drew. These drawings demonstrate the process, the clearing, and questions that I still have…” Her drawing (Figure 11) shows her on a playground and engaging in activities such as rowing and riding a bike. Her playfulness and enlivenment are also demonstrated in the color choices and smiley emoji’s drawn in between the playing figures. Each participant receives the letter from the therapist at the end of the session along with a package of oil pastels and blank journal for the encouragement of their continued process at home.

## Discussion

The REPAT intervention was designed to improve emotion processing and reduce symptoms of depression, pain, and fatigue of cancer survivors. Its theoretical framework is based on the bodymind model of art therapy (Czamanski-Cohen and Weihs, 2016) and findings from the My year after cancer (MYA) study that documented trajectories of persistently high, persistently low and recovering from depressive symptoms of women in the first year after cancer diagnosis (Stanton et al., 2015). The REPAT protocol is unique in its emphasis on emotion processing. While art therapy is described in the literature to be helpful in reducing symptoms of depression and anxiety and improving quality of life (Kievisiene et al., 2020; Bosman et al., 2021), and seen as important in enabling women coping with cancer (and others) to get in touch with and express their emotions (Collie et al., 2006; Puig et al., 2006; Lipson, 2011; von Wietersheim, 2019), and using art for meaning making (Collie et al., 2006) the REPAT protocol, to the best of our knowledge, is the first intervention protocol specifically designed with the goal of increasing awareness, expression and acceptance of emotion, explicitly. Furthermore, the use of body maps is occasionally used in art therapy, as full body tracings for trauma work (Scott and Ross, 2006), as a communication tool for working with pain of cancer patients (Luzzatto et al., 2003), and as an arts based research method (Smit, 2020), this is the first use of body outlines as a way to engage the body, as part of an art therapy protocol.

Along with its strengths, the REPAT protocol should be considered along with its limitations. The REPAT protocol was designed as an 8-week group for the purpose of research; however, our clinical experience shows that BC survivors need much longer than 8 weeks to process the changes that they experience following treatment. How long is not clear, however, Perhaps future studies can examine this. Furthermore, the REPAT protocol is designed for groups, and while we believe in the power of groups, some women may need more individualized attention to become comfortable in exploring difficult emotions.

To date, REPAT has been implemented with women coping with and survivors of BC. The intervention was first piloted in 2015–2016 with ten BC survivors, and the results of the pilot were published (Czamanski-Cohen et al., 2019). Following our successful pilot, we sought funding for a mechanistic study, and were successful in obtaining an RO1 (independent investigator) grant through response to a program announcement with review (PAR)–PAR-14-294- Arts-Based Approaches in Palliative Care for Symptom Management, from the National Institutes of Health Nursing Research institute (NINR; award number R01NR017186). This PAR aimed to support mechanistic clinical studies that would increase understanding of the impact of arts-based approaches in palliative care for symptom management and provide an evidence base for the use of the arts in palliative care for symptom management. It also aimed to support investigation of the biological, physiological, neurological, psychological, and/or sociological mechanisms by which the arts exert their effects on symptom management during and throughout the palliative care continuum. The REPAT study was conducted from 2018 to 2022, and during this time 318 BC survivors were randomized to participate in the REPAT intervention, or a mandala coloring control group. The REPAT study protocol has been published (Czamanski-Cohen et al., 2020) and can be read online, and the results of the REPAT study are currently being analyzed and will be published and presented in the coming years. Links to published work can be found at https://repat.haifa.ac.il/en/.

On October 30th, 2022 we conducted a 1 day conference at the University of Haifa, as a final event of the REPAT study. We presented preliminary results of the study and we gave the participants a forum to ask the team questions about the results and share their experiences with us and the other participants. The most important feedback from the conference attendees was that 8 weeks is not long enough for the support that BC survivors need in the year following primary treatment for BC. Thus, while the REPAT intervention is an 8-week, beneficial protocol for the reduction of symptoms and improving emotion processing, it is likely that women need prolonged support beyond these 8 weeks (GUIDED item nine and ten).

The REPAT intervention is now being adapted in the laboratory for the psychosomatic study of art therapy, at the University of Haifa for individuals coping with inflammatory bowel diseases and for adolescents coping with social anxiety. We hope this intervention protocol will be helpful for art therapy clinicians working with cancer survivors and we would be glad to collaborate with individuals interested in further studying the impact of this intervention.

## Conclusion

The REPAT protocol is an art therapy intervention designed to increase emotion processing and reduce symptoms of depression pain and fatigue of BC survivors. The intervention has been piloted and implemented both online and face to face. It is currently being adapted for and examined with additional populations. While initially designed for research, the REPAT intervention can be implemented in clinical settings, and should possibly be extended to meet the ongoing needs of BC survivors, in the years following coping with cancer and its treatment.

## Data availability statement

The raw data supporting the conclusions of this article will be made available by the authors, without undue reservation.

## Ethics statement

The studies involving human participants were reviewed and approved by the Ethics Committee of the Faculty of Social Welfare and Health Sciences at the University of Haifa. The patients/participants provided their written informed consent to participate in this study. Written informed consent was obtained from the individual(s) for the publication of any potentially identifiable images or data included in this article.

## Author contributions

Both authors listed have made a substantial, direct, and intellectual contribution to the work, and approved it for publication.
