# Supplementary material for: The role of emotion processing in art therapy (REPAT) intervention protocol
Source: Front Psychol. 2023 Jun 29;14:1208901. doi: 10.3389/fpsyg.2023.1208901 (PMC10343444; doi:10.3389/fpsyg.2023.1208901)
Supplement: Supplementary file 1 [file Table_1.docx]

**APPENDIX- The Nature of Emotions**

Emotions are **bodily experiences** that have a role in creating a response to our environment and they inform us if we are directed towards our goals and values. When fundamental emotions are acknowledged and expressed to oneself, they are **transient**. However, when we avoid our emotions, they tend to get stronger and more debilitating and may manifest as physical symptoms or depressive disorders. When emotions are experienced as aversive and thus avoided, they can manifest as emotion driven behaviors, such as rumination, self-criticism.
